# Apparent diffusion coefficient value for estimating clinicohistological factors in bladder cancer including infiltration style and lymphatic invasion

**DOI:** 10.1186/s40064-016-2504-y

**Published:** 2016-06-23

**Authors:** Masaaki Fujimura, Shinichi Sakamoto, Nobuyuki Sekita, Nobuyoshi Takeuchi, Rika Nishikawa, Hiroyoshi Suzuki, Kazuo Mikami, Tomohiko Ichikawa

**Affiliations:** Department of Urology, Chibaken Saiseikai Narashino Hospital, Izumi-cho, Narashino-City, Chiba 275-8550 Japan; Department of Urology, Graduate School of Medicine, Chiba University, 1-8-1 Inohana, Chuo-ku, Chiba-City, Chiba 260-8677 Japan; Department of Urology, Toho University Sakura Medical Center, 564-1 Shimoshizu, Sakura-City, Chiba 285-8741 Japan

**Keywords:** Apparent diffusion coefficient (ADC), Infiltration style (INF), Lymphatic invasion (ly), Bladder cancer

## Abstract

**Objective:**

To evaluate a role of apparent diffusion coefficient (ADC) values measured from diffusion-weighted imaging we investigated its association with clinicopathological tumor characteristics of bladder cancer.

**Materials and methods:**

Diffusion-weighted MRI at 1.5 Tesla using b-values of 0, 1000 s/mm^2^ was taken before transurethral resection by 114 bladder urothelial tumor patients. ADC value was measured and its relationship with pathological factors including T stage, tumor grade, infiltration style (INF) and lymphatic invasion (ly) was analyzed.

**Results:**

Median ADC value was significantly lower in Grade 3 than in Grade 1 (P < 0.001) or in Grade 2 (P = 0.002), in INFb than in INFa (P = 0.004), in INFc than in INFa (P < 0.001), in ly1 than in ly0 (P < 0.001) and lower in T2≦ than in T1≧ (P < 0.001), respectively. Receiver operating curve demonstrated the accuracy of detecting muscle invasive bladder cancer or ly+ by using area under curve (AUC), showing 0.758 and 0.748.

**Conclusion:**

ADC value is likely to serve as a useful biomarker showing clinicopathological characterictics of bladder cancer.

## Background

Bladder cancer is the most common malignancy involving the urological field (Torre et al. 2015). Ultrasonography, computed tomography (CT), and cystoscopy are commonly available for detection of bladder cancer, but are not accurate enough to illustrate histological characteristics, for example, histological grade and tumor stage, which has relationship to recurrence and progression (Sylvester et al. 2006). Diffusion-weighted (DW) MR imaging is helpful to the diagnosis of malignant tumors and an apparent diffusion coefficient (ADC) can reflect tumor characteristics (Takeuchi et al. 2009). ADC value of bladder cancer was lower than that of normal bladder wall and could reflect histological grade and T stage. In addition to these two histological factors, we evaluated whether the two other histological factors, that is, infiltration style (INF) and lymphatic invasion (ly), had significant association with ADC value in bladder cancer. Especially ly is proved to predict not only local and distant recurrence but also adverse overall and disease-specific survival, independent of histological grade and T stage (Lotan et al. 2005). Besides, addition ly to other clinical parameters to nomogram assessing cancer survival or recurrence showed better prediction than TNM classification (Shariat et al. 2010). The purpose of this study was to assess the utility of preoperative ADC value illustrating these four clinico-histological factors, which affect treatment decision and predict metastasis and recurrence of bladder cancer.

## Results

The patients and tumor characteristics are shown in Table 1. Of the 126 patients, 114 had urothelial carcinoma and 9 had the other malignancies; 2 adenocarcinomas, 2 malignant lymphomas, 1 signet ring cell carcinoma, 1 small cell carcinoma, 1 urachal carcinoma, 1 sigmoid carcinoma and 1 undifferentiated carcinoma.

**Table 1:** Patients and tumor characteristics

Variable	Number
Age, years	73 (42–95)*
Gender	
Male	87
Female	27
Tumor size (mm)	20.5 (5–84)*
No. Cancers	2 (1–10)
TNM stage	
T category^#^	
Tis	4
Ta	40
T1	39
T2 or higher	31
N category	
N0	107
N1/2	6/1
M category	
M0	113
M1	1
Histological grade	
Grade 1	9
Grade 2	48
Grade 3	57
Infiltration	
INFa	52
INFb	28
INFc	34
Lymphatic invasion	
ly0	81
ly1	33

* Median (range); ^#^ Pathological T stage was determined by examining TUR specimens

In the 3 patients with a benign lesion, all were diagnosed as erosive cystitis.

We only statistically analyzed tumor characteristics of urothelial carcinoma.

**Fig. 1:**
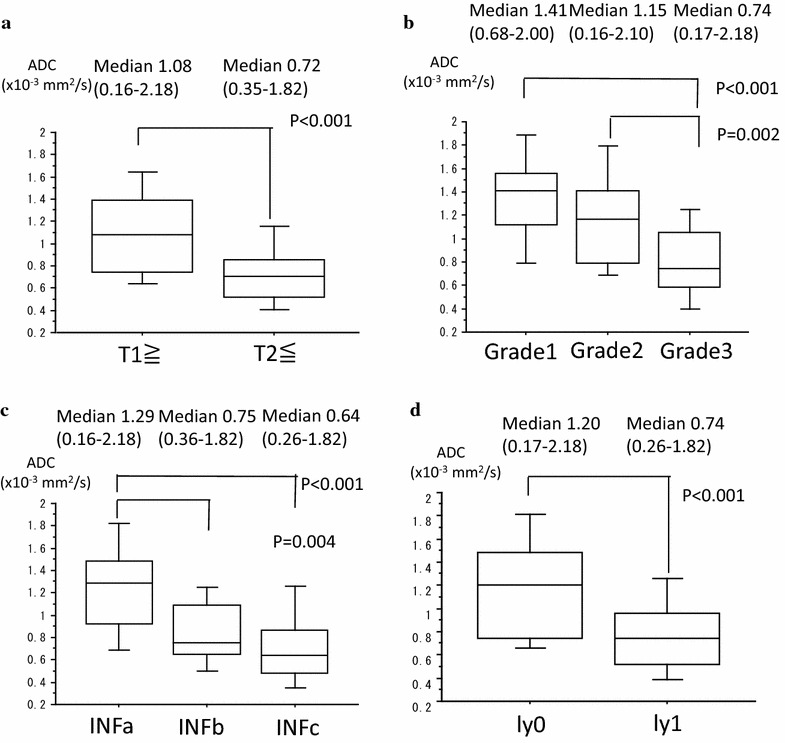
An association of ADC values with, **a** T stage, **b** histological grade, **c** infiltration type (INF) and **d** lymphatic invasion (ly). Boxplots demonstrate the distribution of ADC values according to the T stage, histological grade, INF and ly. The *middle line* in each *box* represents the median, while the lower and upper boundaries of the *boxes* represent lower and upper quartiles (25th and 75th percentiles, respectively)

To investigate whether ADC values in DW-MRI reflect quantitative information on the clinical aggressiveness of bladder cancer, we compared ADC values according to several histologic factors in Fig. 1a–d.

The median ADCs of Grade 1, Grade 2, and Grade 3 tumors were 1.41 × 10^−3^ mm^2^/s, 1.15 × 10^−3^ mm^2^/s, and 0.74 × 10^−3^ mm^2^/s, respectively. The differences in ADC were significant between Grade 1 and Grade 3 (P < 0.001) and between Grade 2 and Grade 3 (P = 0.002).

The median ADCs of INFa, INFb, and INFc tumors were 1.28 × 10^−3^ mm^2^/s, 0.75 × 10^−3^ mm^2^/s, and 0.64 × 10^−3^ mm^2^/s, respectively. The differences in ADC were significant between INFa and INFb (P = 0.004) and between INFa and INFc (P < 0.001).

The median ADCs of ly0 and ly1 were 1.20 × 10^−3^ mm^2^/s and 0.74 × 10^−3^ mm^2^/s, respectively. The differences in ADC were significant between ly0 and ly1 (P < 0.001).

The median ADCs of T1≧ and T2≦ were 1.08 × 10^−3^ mm^2^/s and 0.71 × 10^−3^ mm^2^/s, respectively. The differences in ADC were significant between T1≧ and T2≦ (P < 0.001).

**Fig. 2:**
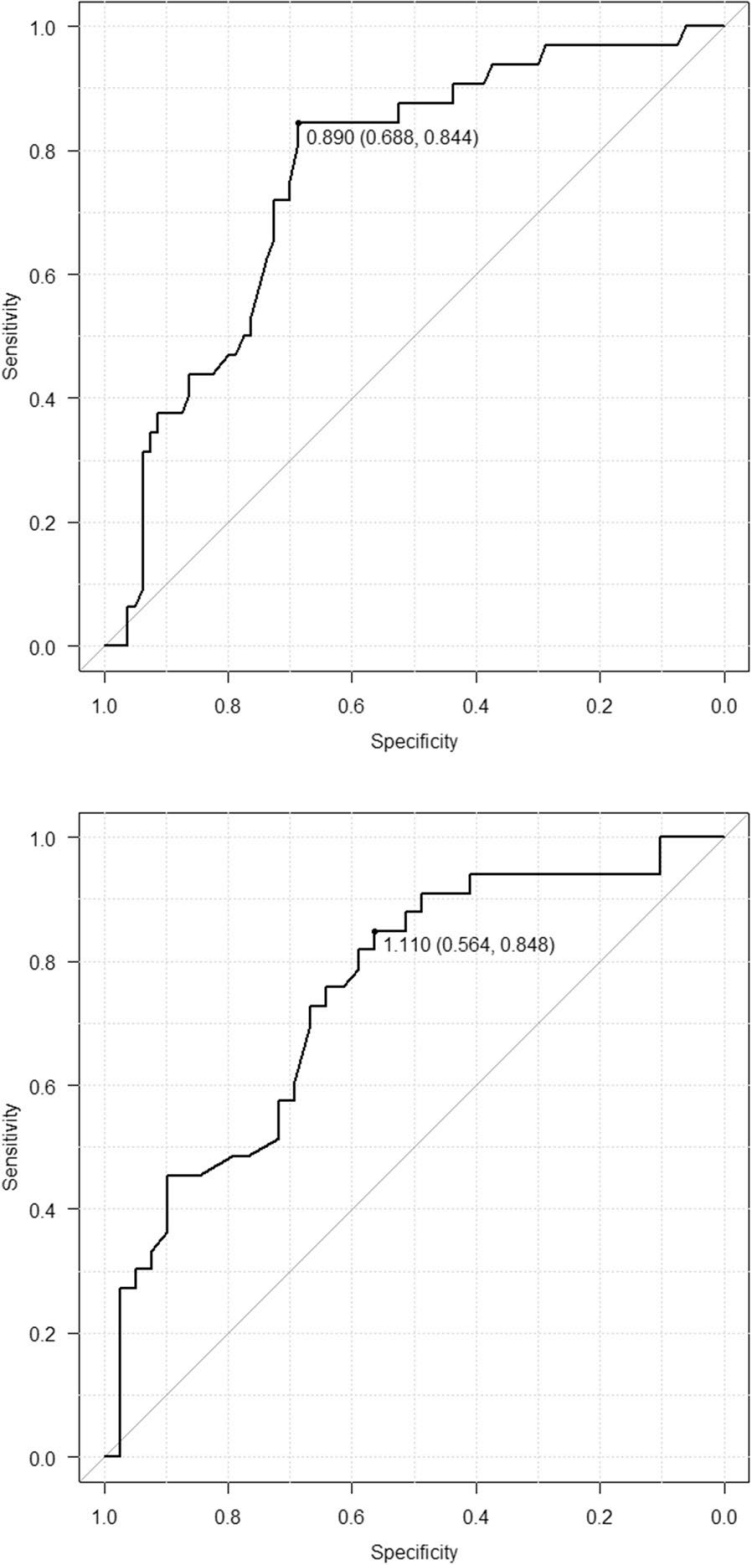
**a** Receiver operating curve (ROC) showed an area under curve (AUC) of 0.758 for predicting muscle invasive bladder cancer (ADC 0.89 × 10^−3^ mm^2^/s; sensitivity 68.8 %, specificity 84.4 %). **b** 0.747 for predicting lymphvascular invasion (ly+) (ADC 1.11 × 10^−3^ mm^2^/s; sensitivity 56.4 %, specificity 84.8 %)

Diagnostic performance of ADC values in differentiating not only MIBC and NMIBC but also ly+ and ly− are shown in Fig. 2a, b.

The cut-off value in the receiver operating characteristic (ROC) curve for differentiating MIBC and NMIBC according to ADC values was found to be 0.89 × 10^−3^mm^2^/s, of which area under curve (AUC) was 0.758. Based on these values, the sensitivity was 68.8 % and the specificity was 84.4 %. The value for differentiating ly+ versus ly− according to ADC values was found to be 1.11 × 10^−3^ mm^2^/s, of which AUC was 0.747. Based on these values, the sensitivity was 56.4 % and the specificity was 84.8 %.

## Discussion

Diffusion illustrates the random motion of water molecules in the tissue, which is called “Brownian motion”. Currently from DW images the measurement of ADC values can be the quantitative assessment for this phenomenon. Malignancies tend to include a larger cell diameter, higher cellularity than normal tissue. Therefore water diffusion is disturbed, which lead to lower ADC value (Padhani et al. 2009). ADC value provided us to qualitative evaluation of malignant tissues.

ADC value of bladder cancer was lower than that of normal bladder tissue. The mean ADC value of G3 tumors was significantly lower than that of either G1 or G2 tumors, and all G3 tumors showed an ADC value less than 1.0 × 10^−3^ mm^2^/s (Takeuchi et al. 2009), which was almost compatible to our results. Tumor grade is influenced by several pathological factors reflecting on cellular density, the ADC value can predict the histologic grade of bladder cancer. Besides, though our result did not show significant difference between G1 and G2 tumor, ADC value of G3 tumor was lower than that of both G1 and G2. Whether tumor include G3 component or not affects following treatment, because G3 tumor in NMIBC often requires additional treatment; not only intravesical instillation of anti-cancer drug or BCG but also second look TUR, in case of NMIBC, therefore ADC value can be a preoperative helpful tool predicting following treatment. Histological grade and T stage affected cancer-specific survival in bladder cancer (Sugahara et al. 1999), therefore preoperative quantitative assessment of ADC value may predict survival benefit and avoid unnecessary procedure in bladder cancer.

INF illustrates the style of tumor infiltration to deep layer of bladder and ly demonstrates tumor invasion to lymph vessel. Both histologic factors have relationship to tumor aggressiveness and potential of metastasis or invasion to adjacent organ. Infiltration of the vascular and/or lymphatic structures by tumor cells is an essential process in tumor dissemination (Padera et al. 2002; Alitalo et al. 2004; Kikuchi et al. 2009; Akao et al. 2008). Malignant cells invade the lymphovascular space, proliferate, and then permeate the local lymphatics or spread more widely (Alexander-Sefre et al. 2003). Lymphatic invasion is independently associated with overall survival, cause-specific survival, and local and distant recurrence in patients with negative lymph nodes at radical cystectomy (Lotan et al. 2005; Kim et al. 2014). The current study demonstrated that lower ADC value was related to stronger degree of tumor infiltration and the presence of lymphatic invasion. Unfortunately our study didn’t show lower ADC value was directly related to survival or metastatic risk (data not shown), but ADC value may predict survival or metastatic risk if more data is accumulated. Although our result demonstrated that INFb was not significantly higher than INFc because of pathological difficulty in discrimination between INFb and INFc. But it has to be cautioned that more advanced T stage, higher grade, stronger infiltration and lymphatic invasion may express the phenomenon of tumor aggressiveness simultaneously. A prospective validation study is awaited in order to assess availability of measuring ADC value in predicting tumor aggressiveness.

Our study have several numbers of limitation. First, inflammatory changes before MR imaging might affect ADC evaluation in some cases (Barentsz et al. 1996), because inflammatory cells disturbe water molecule’s diffusion. Second, all the radiologic result did not match pathologic result because we could not identify all the cut surfaces on surgical specimens which was seen on MR images. Besides specimen derived from TUR were cut into small pieces, which lead to make evaluation of INF or ly more difficult. Third, ADC value is reflected on MRI parameters including the coil systems, scanning systems, FOV, repetition time, echo time, matrix, b-values and many other parameters (Sasaki et al. 2008), therefore cut-off ADC value determined in our study may not suitable at the other institutional imaging protocols. In order to revise the difference, relative parameters, such as ADC ratios, calculated as ADC value in tumor/ADC value in adjacent structures (normal bladder wall or obturator internus muscle), would be more reliable in case of comparison among different institution. Finally, there may be selection bias in this study. Small or flat tumors like carcinoma in situ, which were undetectable on MRI were eliminated from current study. DW imaging requires enough space for assessment, compared with normal MRI imaging.

## Conclusion

Our results demonstrated that DW imaging with ADC measurement is reliable method for clinicopathological tumor aggressiveness of bladder. In addition ADC value may supplement other currently applied parameters for predicting tumor behavior and making treatment decisions. However, further studies with larger series are needed to validate a more efficient use of this technique and a wider clinical application.

## Methods

### Patients

Our hospital’s institutional review board approved this study. The requirement for patients’ informed consent was waived for this retrospective study.

Between April 2009 and November 2013, 126 patients with cystoscopy-proven bladder tumors underwent MRI, including both DW-MRI and T2-weighted magnetic resonance imaging (T2W-MRI) protocols, before transurethral resection in a retrospective study investigating the potential role of DW-MRI and/or ADC value as a biomarker for bladder cancer. Patients with histologically confirmed bladder cancer were eligible for analysis.

### Imaging protocol

Multi-sequence MRI was carried out with a 1.5-Tesla imager (Toshiba, Japan) under free breathing with 4-channel sensitivity encoding body coil after a 4–6 h fast and voiding 1 h before the examination. The imaging parameters for DW-MRI with single-shot echo planar imaging sequence were set as follows: repetition time, 8000 ms; echo time, 80 ms; matrix, 128 × 128; field of view, 38 cm; acquisition time, 220 s; slice thickness, 5 mm; interslice gap, 0.5 mm; number of slices, 24; number of excitations, 8; bandwidth, 1302 Hz per pixel; two different diffusion gradient b-values (b = 0, 1000 s/mm^2^); fat suppression, spectral pre-saturation inversion recovery. T2W-MRI, the parameters were set as follows: repetition time, 4000 ms; echo time, 90 ms; matrix, 256 × 38; field of view, 20 cm; acquisition time, 180 s; slice thickness, 4 mm; interslice gap, 0.4 mm; number of slices; 24; number of excitations, 3; bandwidth, 122 Hz per pixel.

### Detection of bladder cancer by DW-MRI and T2 W-MRI

**Fig. 3:**
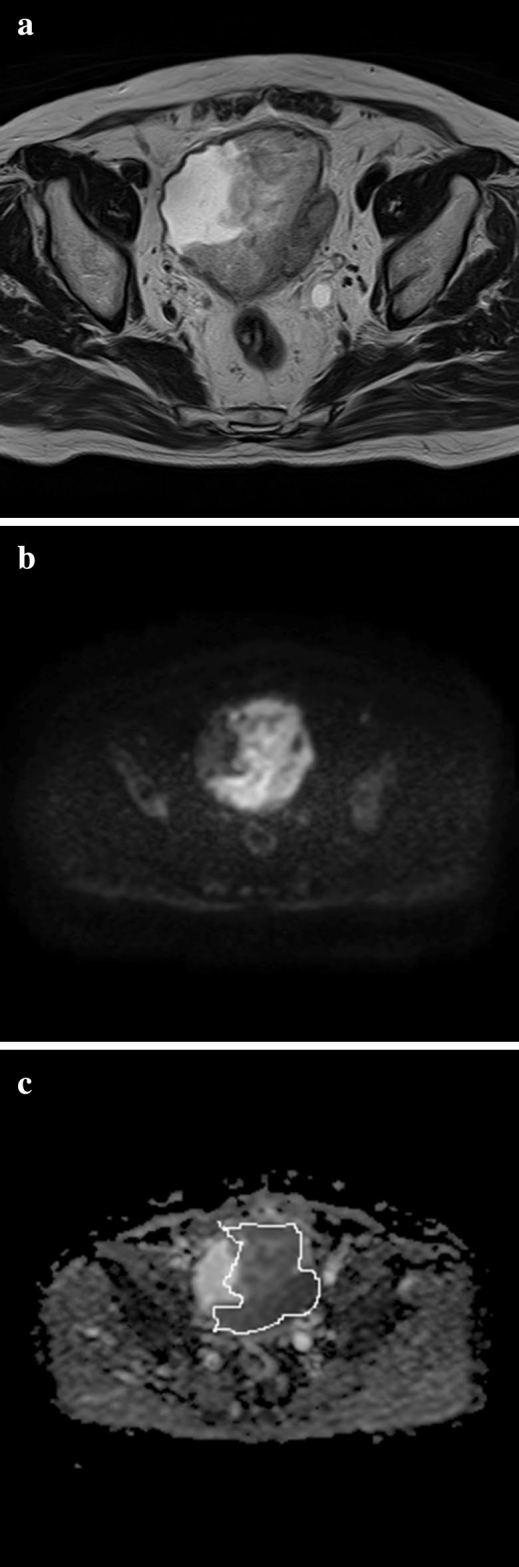
MR images of 80-year-old man with histologically confirmed muscle-invasive bladder tumor (MIBC). **a** Tumor shows the thickened muscle layer focally disrupted on transverse T2WMRI. **b** Tumor shows high intensity area without submocosal components on transverse DW-MRI (**b**; 0, 1000 s/mm^2^). **c** ADC values or tumor, measured within an ROI is 0.94 × 10^−3^ mm^2^/s

Detection of bladder cancer on MRI was defined as a mass with high signal intensity (SI) arising in the normal bladder wall with low SI (Fig. 3).

All image sets of DW-MRI and T2W-MRI were independently reviewed by two radiologists (I.M., I.H.; both have 20 years of experience, respectively) who were blinded to the TUR and histopathological findings. The reviewer assessed intravesical tumor location and T stage by reviewing images on transverse planes. Patients with hypersignal lesions with a b-value of 1000 s/mm^2^ on DW-MRI or those showing mass formation on T2W-MRI were diagnosed with bladder cancer.

### TUR findings

The numbers, sizes (maximum diameter), and intravesical locations of bladder tumors were recorded.

### TNM classification

Nodal and metastatic evaluation was performed according to TNM Classification of Malignant tumours 7th edition.

### Histopathological findings

Histological grade, lymphatic invasion, vessel invasion, schirrous change and pathological T stage were determined according to the 2004 World Health Organization/International Society of Urological Pathology classification and the 2009 TNM system, respectively. In patients with multiple tumors, each patient’s T stage and histological grade reflects the conditions of that patient’s highest-stage index tumor. Tumors were classified into three grades: G1, the least degree of anaplasia; G2, an intermediate degree of anaplasia: and G3, severe anapalsia. Histological invasion form was also classified into three forms: INFa, tumor growth shows expandingly and tumor is clearly divided between benign tissue; INFb, tumor shows intermediate growth between INFa and INFc: INFc, tumor invasion in the deepest point shows cellularity or in micronodule form. A pathologist with 30-year of experience in urogenital pathology was responsible for performing all pathological examinations.

### ADC measurement

To quantitatively analyze the degree of diffusion, ADC values were calculated in tumors correctly detected by two radiologists on DW-MRI. ADC maps of lesions were reconstructed with the two different diffusion gradient b-values at a workstation (Toshiba, Japan). Regions of interests (ROIs), maximally covering but not extending beyond the edges of the lesions, were identified for measurement of ADC values on transverse ADC maps. In case of visible tumors in multiple slices on the ADC map, ROIs were placed on each slice; from base to top of tumor. Among all slices minimum ADC value was adopted to statistic anlysis. In case of multiple tumors in one slice, ADC value of all tumors was measured, minimum ADC value was then analyzed. ROIs were set to be at least 20 mm^2^ in order to minimize the influence of potential motion artifacts (Fig. 3).

### Statistical analysis

Differences in ADC values according to T stage or ly were assessed using the Mann–Whitney U test. In addition, differences in ADC values according to INF or Grade were Scheffe test. Predictive accuracy was assessed using area under the receiver operating characteristics curve. All P values < 0.05 were considered statistically significant. R version 3.0.2 was used for all statistical analyses.

## Abbreviations

ADC: apparent diffusion coefficient
DWI: diffusion-weighted imaging
INF: infiltration style
ly: lymphatic invasion
AUC: area under curve
NMIBC: non-muscle-invasive bladder cancer
MIBC: muscle-invasive bladder cancer
TUR: transurethral resection
CT: computed tomography
ROI: region of interest
